# Effects of Cilostazol on Angiogenesis in Diabetes through Adiponectin/Adiponectin Receptors/Sirtuin1 Signaling Pathway

**DOI:** 10.3390/ijms232314839

**Published:** 2022-11-27

**Authors:** Shih-Ya Tseng, Hsien-Yuan Chang, Yi-Heng Li, Ting-Hsing Chao

**Affiliations:** 1 Division of Cardiology, Department of Internal Medicine, National Cheng Kung University Hospital, College of Medicine, National Cheng Kung University, Tainan 704, Taiwan; 2 Department of Biological Science, National Sun Yat-sen University, Kaohsiung 804, Taiwan; 3 Institute of Clinical Medicine, College of Medicine, National Cheng Kung University, Tainan 704, Taiwan; 4 Health Management Center, National Cheng Kung University Hospital, Tainan 704, Taiwan

**Keywords:** cilostazol, angiogenesis, adiponectin, sirtuin, adiponectin receptor, hyperglycemia

## Abstract

Cilostazol is an antiplatelet agent with vasodilating effects that functions by increasing the intracellular concentration of cyclic adenosine monophosphate. We have previously shown that cilostazol has favorable effects on angiogenesis. However, there is no study to evaluate the effects of cilostazol on adiponectin. We investigated the effects of cilostazol on angiogenesis in diabetes in vitro and in vivo through adiponectin/adiponectin receptors (adipoRs) and the sirtuin 1 (SIRT1)/AMP-activated protein kinase (AMPK) signaling pathway. Human umbilical vein endothelial cells (HUVECs) and human aortic smooth muscle cells (HASMCs) were cocultured under high glucose (HG) conditions. Adiponectin concentrations in the supernatants were significantly increased when HASMCs were treated with cilostazol but not significantly changed when only HUVECs were treated with cilostazol. Cilostazol treatment enhanced the expression of SIRT1 and upregulated the phosphorylation of AMPK in HG-treated HUVECs. By sequential knockdown of adipoRs, SIRT1, and AMPK, our data demonstrated that cilostazol prevented apoptosis and stimulated proliferation, chemotactic motility, and capillary-like tube formation in HG-treated HUVECs through the adipoRs/SIRT1/AMPK signaling pathway. The phosphorylation of downstream signaling molecules, including acetyl-CoA carboxylase (ACC) and endothelial nitric oxide synthase (eNOS), was downregulated when HUVECs were treated with a SIRT1 inhibitor. In streptozotocin-induced diabetic mice, cilostazol treatment could improve blood flow recovery 21–28 days after inducing hindlimb ischemia as well as increase the circulating of CD34^+^CD45^dim^ cells 14–21 days after operation; moreover, these effects were significantly attenuated by the knockdown of adipoR1 but not adipoR2. The expression of SIRT1 and phosphorylation of AMPK/ACC and Akt/eNOS in ischemic muscles were significantly attenuated by the gene knockdown of adipoRs. Cilostazol improves HG-induced endothelial dysfunction in vascular endothelial cells and enhances angiogenesis in diabetic mice by upregulating the expression of adiponectin/adipoRs and its SIRT1/AMPK downstream signaling pathway.

## 1. Introduction

Cilostazol, a phosphodiesterase type 3 inhibitor, is an antiplatelet agent and is indicated for peripheral artery disease patients suffering from intermittent claudication [1,2]. This drug increases cyclic adenosine monophosphate (cAMP) levels by inhibiting PDE and has antiplatelet and vasodilatory effects. Cilostazol was also shown to have pleiotropic effects on endothelial cells (ECs), including protection from apoptotic cell death [3], increased endothelial nitric oxide synthase (eNOS) activity [4], stimulation of the release of angiogenic factors [5], and improvement of endothelial function [6]. In addition, several studies have demonstrated that cilostazol has beneficial effects on endothelial progenitor cells [7,8] and vascular angiogenic effects in a murine model of hindlimb ischemia [5]. Our study also showed that the vascular angiogenic effects of cilostazol are modulated by adenosine monophosphate-activated protein kinase (AMPK)/acetyl-coenzyme A carboxylase (ACC) and probably by the subsequent Akt/eNOS pathway [6]. Therefore, AMPK plays a pivotal role in the effects of cilostazol on angiogenesis, but the detailed mechanism of action remains unclear.

Adiponectin is a secretory protein produced predominantly by adipose tissue [9] and has beneficial effects on insulin sensitivity, lipid metabolism, and vasoprotective properties [10,11], which are mainly mediated by adiponectin receptor 1 (adipoR1), adiponectin receptor 2 (adipoR2), and T-cadherin. AdipoR1 is abundantly expressed in skeletal muscle and is associated with the activation of the AMPK pathway [12,13], whereas adipoR2 is abundantly expressed in the liver and is associated with peroxisome proliferator-activated receptor alpha (PPARα) ligand activity [14,15]. Several clinical studies have indicated that hypoadiponectinemia is associated with endothelial dysfunction [16,17,18], and animal studies have also shown that adiponectin has anti-atherosclerotic [19,20] and angiogenic [21] effects. In addition, previous studies showed that the effects of adiponectin were mediated by the sirtuin 1 (SIRT)/AMPK pathway [22,23,24]. Thus, by activating the SIRT/AMPK axis, adiponectin also plays an important role in angiogenesis. Notably, adiponectin is not only produced by adipose tissue but also derived from vascular smooth muscle cells (SMCs) [25] and plays a critical function in the contractile phenotype [26]. Only one clinical study showed that cilostazol increases adiponectin levels [27], and the mechanism is unclear. However, to the best of our knowledge, there has been no study evaluating the effects of cilostazol on adiponectin and its receptors in terms of angiogenesis and its mechanism.

In the current study, we tried to fill up the knowledge gap about how cilostazol works on adiponectin, adipoR1, adipoR2, and the downstream signaling pathway. We hypothesized that cilostazol prevents endothelial dysfunction in vascular ECs and enhances angiogenesis by upregulating adiponectin secretion from SMCs, and to test this we investigated the effects of cilostazol on angiogenesis in vitro and in vivo through adiponectin/adipoRs and the SIRT1/AMPK signaling pathway.

## 2. Results

### 2.1. Cilostazol Increased Adiponectin Levels in ECs by Stimulating Their Production from SMCs

**Figure 1:**
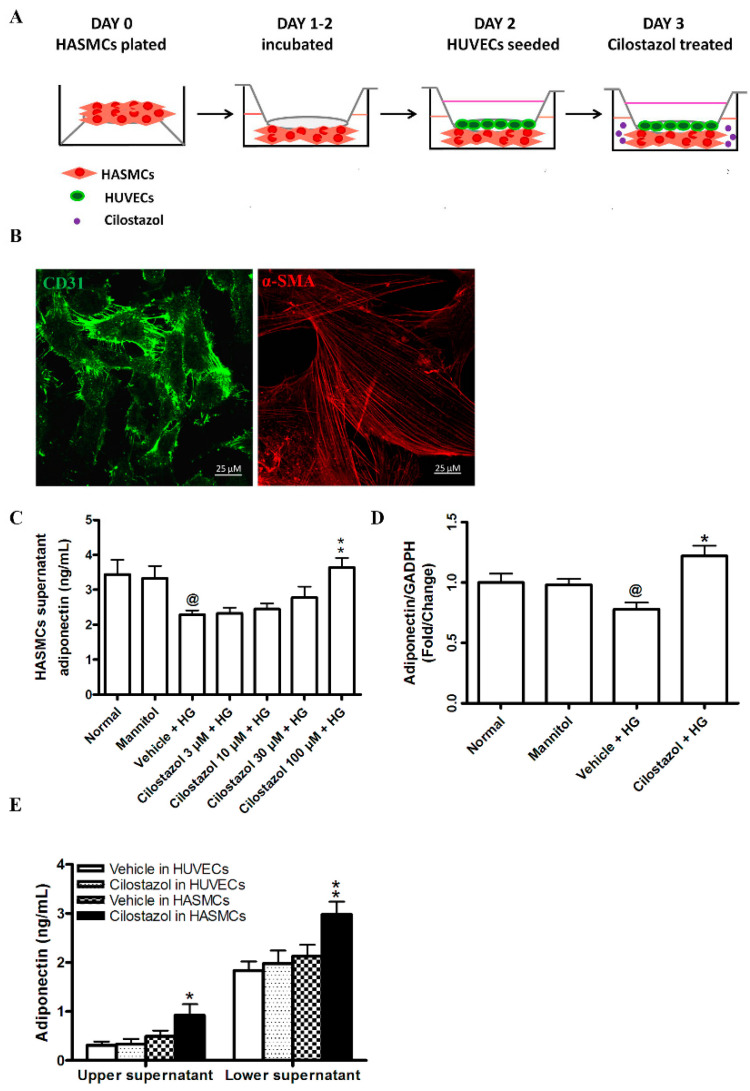
Cilostazol increased adiponectin levels in HUVECs by stimulating HASMC production under HG conditions. (**A**) Schematic diagram of the Transwell coculture system. HUVECs were seeded in the upper chamber, and HASMCs were cultured in the lower chamber under HG (25 mmol/L) conditions. (**B**) Representative images of immunostaining of HUVECs with CD31 (green) and HASMCs with α-SMA (red). Scale bar: 25 μM. (**C**) Quantification of adiponectin levels in the supernatants of the lower insert using ELISAs. Mannitol was used as an osmolality control. (**D**) Quantification of adiponectin expression in HASMCs using real-time PCR. (**E**) Quantification of adiponectin levels in the cilostazol-treated coculture system under HG conditions using ELISAs. The results are represented as the mean ± S.E.M. from three independent experiments. @ *p* < 0.05 versus the normal group; * *p* < 0.05 and ** *p* < 0.01 versus the vehicle group.

After the seeding and incubation of human aortic smooth muscle cells (HASMCs), human umbilical vein endothelial cells (HUVECs) were cocultured under high glucose (HG) conditions and then treated with cilostazol (Figure 1A). Both HASMCs and HUVECs were confirmed using immunofluorescence (Figure 1B). Under HG conditions, the concentrations of adiponectin in SMC supernatants were significantly decreased compared with those in the mannitol group without HG (2.29 ± 0.12 vs. 3.33 ± 0.35 ng/mL). These results indicated the attenuated secretion of adiponectin from HASMCs under HG conditions. However, after treatment with 100 µM of cilostazol under HG conditions, adiponectin levels were significantly increased (3.64 ± 0.27 vs. 2.29 ± 0.12 ng/mL, Figure 1C,D). This finding implied that cilostazol reverses the HG-induced reduction in adiponectin secretion from HASMCs. In addition, the adiponectin concentrations in the HUVEC supernatants were significantly increased in a dose-dependent manner when HASMCs were treated with cilostazol but not significantly changed when only HUVECs were treated with cilostazol (Figure 1E). Accordingly, cilostazol increased the adiponectin concentrations in the supernatants of the Transwell system cultured with HUVECs via paracrine effects from SMCs.

### 2.2. Cilostazol Increased Angiogenic Functions by Upregulating the Expression of Adiponectin Receptors and Enhancing Adiponectin Released from SMCs by Binding to Adiponectin Receptors in HUVECs

**Figure 2:**
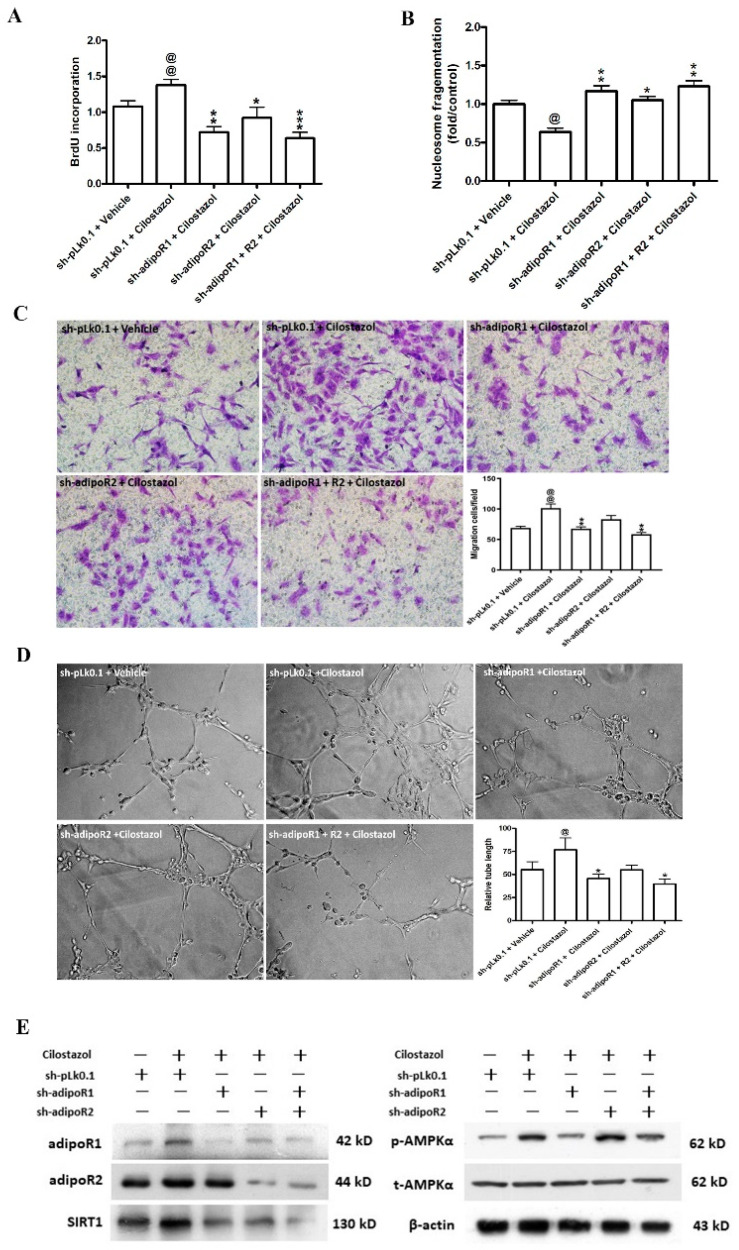
Knockdown of adiponectin receptors attenuated the angiogenic functions of cilostazol in HG-treated HUVECs. AdipoR1 shRNA, adipoR2 shRNA, and control shRNA (pLk0.1) lentivirus were used to infect HUVECs. Then, the Transwell coculture system was incubated under HG conditions in the presence or absence of cilostazol (100 μM) on the undersides of the inserts for 24 h. (**A**) Quantification of BrdU incorporation. (**B**) Quantification of DNA fragmentation by cell death using ELISAs. (**C**) Representative images (×200) of migrating cells stained with crystal violet and their quantification. (**D**) Representative photomicrographs (×200) of the tube formation assays and quantitation of branching points. (**E**) Effects of adiponectin receptor knockdown on cilostazol-induced AMPK phosphorylation and SIRT1 activity. Representative images of Western blot analysis of cell lysates from HUVECs. The results are represented as the mean ± S.E.M. from triplicate wells in three experiments. @ *p* < 0.05 and @@ *p* < 0.01 versus the vehicle group; * *p* < 0.05, ** *p* < 0.01, and *** *p* < 0.001 versus the sh-pLk0.1 group treated with cilostazol (100 μM).

To investigate how HUVECs were affected after coculturing with HASMCs treated with cilostazol, we transfected sh-adipoR1 and sh-adipoR2 to knock down adiponectin receptors in HUVECs. Our data showed that cilostazol significantly stimulated cell proliferation (1.38 ± 0.08 vs. 1.08 ± 0.08), migration (81.8 ± 12.8 vs. 59.6 ± 9.3 cells/field), and decreased nucleosome fragmentation in HG-treated HUVECs. These effects of cilostazol were attenuated after the knockdown of the adiponectin receptors (Figure 2A–C). With regard to vascular tube formation, HG-treated HUVECs failed to form networks but the capillary-like tube network was more enriched and longer when HUVECs were treated with cilostazol (relative tube length: 76.8 ± 12.8 vs. 55.3 ± 8.6 μm/field). However, after the knockdown of adipoR1, the tube length was significantly shortened (45.6 ± 4.9 vs. 76.8 ± 12.8 μm/field, Figure 2D). In addition, after the knockdown of adipoRs, the stimulatory effects of cilostazol on the expression of the downstream signaling molecules, specifically SIRT1 and p-AMPKα, was also decreased (Figure 2E). Thus, adipoRs play an important role in modulating the angiogenic effects of cilostazol by increasing adiponectin secretion from HASMCs.

### 2.3. Cilostazol Enhanced SIRT/AMPK Signaling in HUVECs

**Figure 3:**
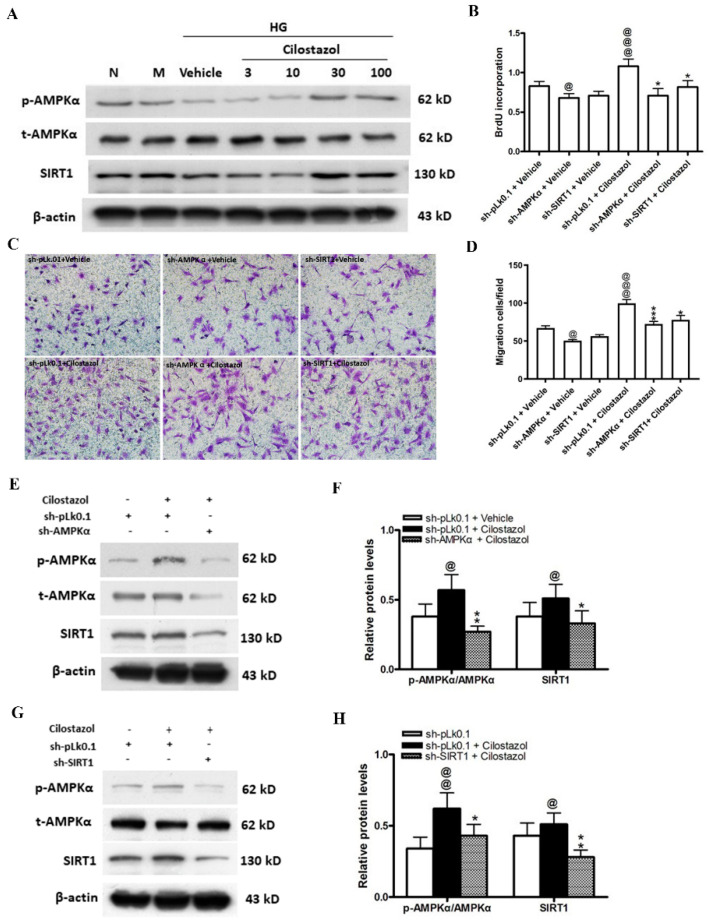
Cilostazol upregulated the AMPK/SIRT axis in HUVECs pretreated with HG. (**A**) Cilostazol stimulated the phosphorylation of AMPKα and SIRT1 activity in a dose-dependent manner, with peak effects at 100 μM of cilostazol. (**B**) Quantification of BrdU incorporation data of HUVECs transfected with AMPKα or SIRT1 shRNA and cocultured with or without cilostazol-treated HASMCs for 24 h. (**C**) Representative images (×200) of migrating cells stained with crystal violet. (**D**) Quantification of migrating cells. (**E**–**H**) Western blot assays of AMPKα and SIRT1 in HUVECs transfected with AMPKα shRNA (**E**), SIRT1 shRNA (**G**), or nontargeting pLk0.1 shRNA and cocultured with HASMCs treated with vehicle or cilostazol. (**F**,**H**) Quantification of Western blot data. The results are represented as the mean ± S.E.M. from three independent experiments performed in triplicate. @ *p* < 0.05, @@ *p* < 0.01, and @@@ *p* < 0.001 versus the sh-pLk01 + vehicle group; * *p* < 0.05, ** *p* < 0.01, and *** *p* < 0.001 versus the sh-pLk0.1 group treated with cilostazol (100 μM).

We next investigated the expression of the downstream pathway components of SIRT/AMPK in HUVECs cocultured with cilostazol-treated HASMCs. As expected, HG conditions decreased SIRT1 and the phosphorylation of AMPKα; however, cilostazol treatment restored the phosphorylation of both molecules (Figure 3A). The knockdown of SIRT or AMPKα attenuated the stimulating effects of cilostazol on cell proliferation and migration in HG-treated HUVECs (Figure 3B–D). When AMPKα was knocked down, the stimulating effects of cilostazol on the SIRT1 protein was diminished (Figure 3E,F). However, the phosphorylation of AMPK was also significantly decreased (0.43 ± 0.08 vs. 0.62 ± 0.11) after the SIRT knockdown even when HUVECs were cocultured with cilostazol-treated HASMCs (Figure 3G,H). This finding indicates that SIRT and AMPK mutually act as upstream and downstream signaling molecules, and this axis plays an important role in cilostazol-induced angiogenesis.

### 2.4. Contributions of SIRT to the Cilostazol-Induced Angiogenic Effects

**Figure 4:**
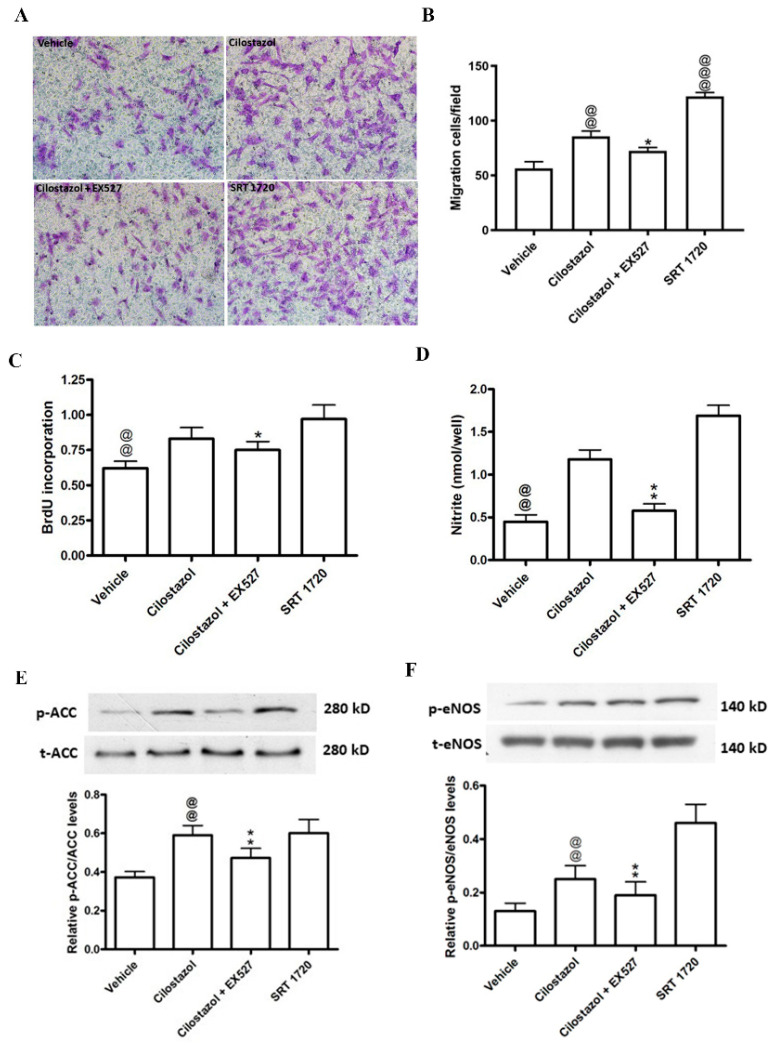
Contributions of SIRT to the cilostazol-induced angiogenic effects on HUVECs pretreated with HG. HUVECs were cultured for 3 days in serum-free M199 containing EX527 (10 μM) and SRT 1720 (0.1 μM) and then cocultured with HASMCs treated with vehicle or cilostazol. (**A**) Representative images (×200) of migrating cells stained with crystal violet. (**B**) Quantification of migrating cells. (**C**) Quantification of BrdU incorporation. (**D**) Nitrite production in HUVECs. (**E**,**F**) Cell lysates were detected for ACC, p-ACC (**E**), eNOS and p-eNOS (**F**) using Western blot analysis. The results are represented as the mean ± S.E.M. from three independent experiments performed in triplicate. @@ *p* < 0.01 and @@@ *p* < 0.001 versus the vehicle group; * *p* < 0.05 and ** *p* < 0.01 versus the cilostazol (100 μM) group.

To clarify the downstream pathway of adiponectin secreted from cilostazol-treated HASMCs, we used a SIRT enhancer, SRT 1720, and its inhibitor, EX527, for the subsequent experiments. The effects of cilostazol on cell migration, proliferation, and nitric oxide production were decreased after EX527 treatment, whereas SRT1720, which served as a positive control, had similar effects to cilostazol (Figure 4A–D). Furthermore, cilostazol increased the phosphorylation of ACC (0.59 ± 0.05 vs. 0.37 ± 0.03) and eNOS (0.25 ± 0.05 vs. 0.13 ± 0.03), while EX527 attenuated these effects (Figure 4E,F). In contrast, after treatment with SRT 1720, the phosphorylation of ACC and eNOS was enhanced (p-ACC: 0.60 ± 0.07 vs. 0.37 ± 0.03; p-eNOs: 0.46 ± 0.07 vs. 0.13 ± 0.03, Figure 4E,F). This result demonstrated that the vascular angiogenic effects of cilostazol are modulated by SIRT and its downstream signaling pathway is the ACC/eNOS axis.

### 2.5. The Angiogenic Effects of Cilostazol were Mediated by AdipoRs In Vivo

**Figure 5:**
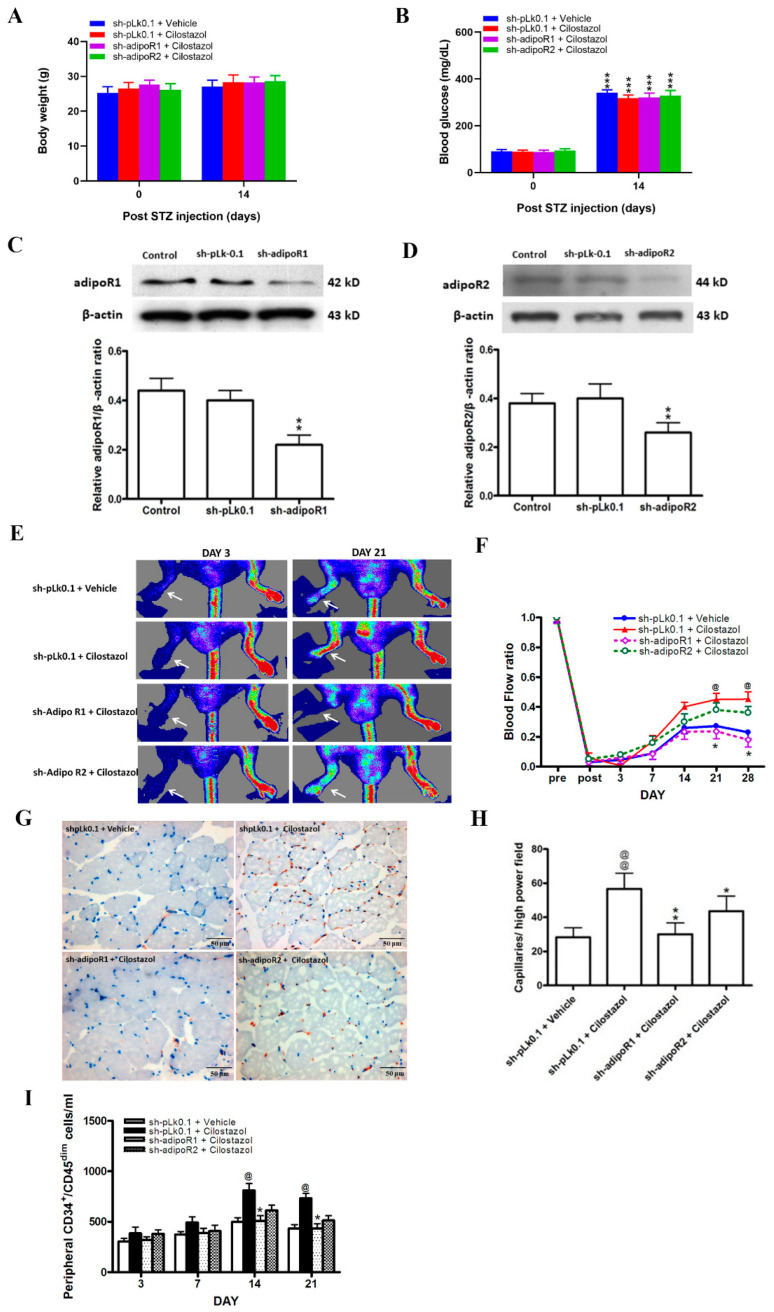
AdipoRs play a pivotal role in the cilostazol-mediated adiponectin angiogenesis in STZ-induced diabetic mice undergoing hindlimb ischemia. STZ-induced diabetic mice were transfected with adipoR1 sh-RNA or adipoR2 sh-RNA to knock down adipoRs. Animals were then injected with cilostazol (30 mg/kg) or vehicle twice daily for one week. (**A**) Body weight and (**B**) plasma glucose levels in the adipoR1 or adipoR2 knockdown mice. *** *p* < 0.001 compared with day 0. The protein expression levels of adipoR1 (**C**) and adipoR2 (**D**) were significantly decreased by plasmid transfection. ** *p* < 0.05 versus the control group. (**E**) Laser Doppler analysis of blood perfusion in the hindlimbs of the mice (indicated by arrows) on days 3, 7, 14, 21 and 28. (**F**) Quantification of the blood flow ratio. (**G**) Representative photomicrographs showing CD31 (red) and nuclei (blue) in ischemic muscle four weeks after femoral artery ligation. Scale bar: 50 μM. (**H**) Quantification of CD31-positive cells. (**I**) Quantification of circulating CD34^+^/CD45^dim^ cells in mice analyzed using flow cytometry. The results are represented as the mean ± S.E.M. from five mice per group. @ *p* < 0.05 and @@ *p* < 0.01 versus the sh-pLk0.1 and vehicle-treated mice; * *p* < 0.05 and ** *p* < 0.01 versus the sh-pLk0.1 and cilostazol-treated mice.

We next used streptozotocin (STZ)-induced diabetic mice treated with cilostazol after inducing hindlimb ischemia to investigate the effects of cilostazol and adipoRs on angiogenesis. After the knockdown of adipoR1, the body weights of the mice were not different (Figure 5A). Diabetes was successfully induced with very consistent plasma glucose levels across four groups (Figure 5B). Western blot analysis confirmed that adipoRs were successfully knocked down (Figure 5C,D). In the sh-adipoR1 group, the recovery of the blood flow ratio measured in the ipsilateral limb versus the contralateral limb was significantly lower than those of the other groups 21–28 days after surgery (day 21: 0.24 ± 0.04 vs. 0.45 ± 0.04; day 28: 0.18 ± 0.05 vs. 0.45 ± 0.05, Figure 5E,F). However, capillary density was significantly increased in cilostazol-treated mice; this effect was attenuated while adipoRs were knocked down, especially adipoR1 (Figure 5G,H). The circulating number of CD34^+^CD45^dim^ cells was increased in the cilostazol group compared with the control group, and this effect was attenuated in the sh-adipoR1 group (Figure 5I). Taken together, our data implied that the angiogenic effects of cilostazol are mediated by adiponectin/adipoRs, mainly adipoR1.

**Figure 6:**
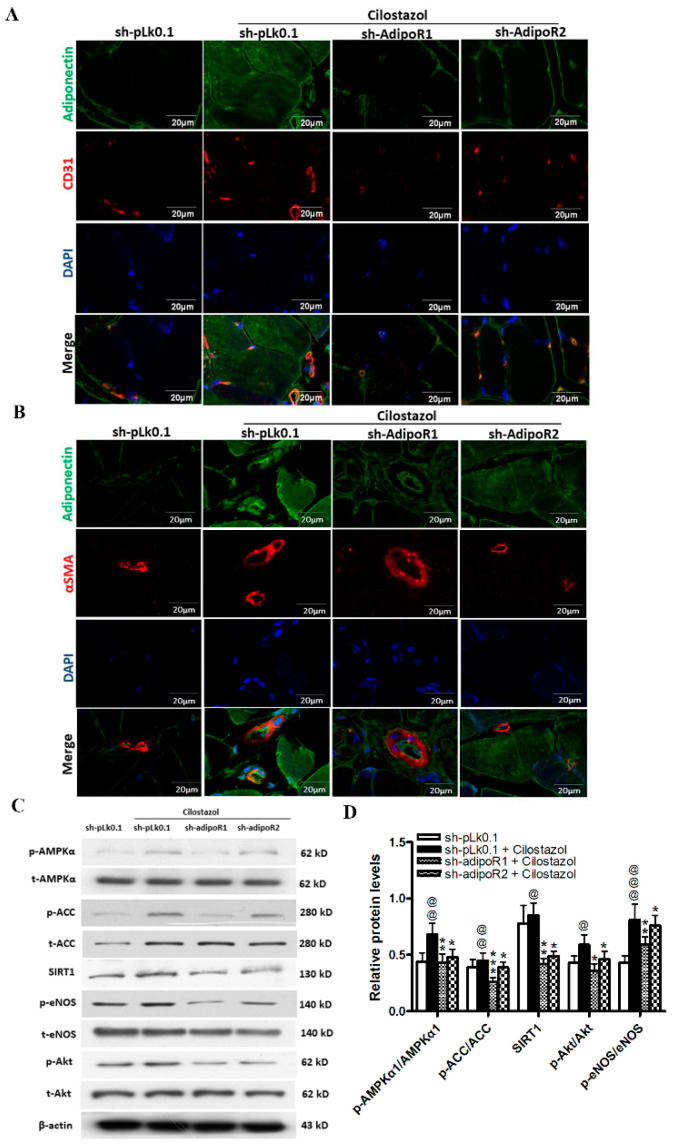
The beneficial effects of cilostazol on angiogenesis were initially mediated by enhancing adiponectin expression in a hindlimb ischemia model of STZ-induced diabetic mice. (**A**,**B**) Representative photomicrographs showing colocalization of adiponectin (green), nuclei (DAPI, blue), and ECs ((**A**), CD31, red) or SMCs ((**B**), α-SMA, red) in ischemic muscle four weeks after femoral artery ligation. Scale bar: 30 μM. (**C**,**D**) Western blot assays and quantification of phosphorylation of AMPKα, ACC, Akt, eNOS, and SIRT1 activity in muscles. The results are represented as the mean ± S.E.M. from five mice per group. @ *p* < 0.05, @@ *p* < 0.01, and @@@ *p* < 0.001 versus the vehicle group; * *p* < 0.05, ** *p* < 0.01, and *** *p* < 0.001 versus the sh-pLk0.1 group treated with cilostazol (100 μM).

In ischemic muscle four weeks after femoral artery ligation, colocalization of immunofluorescence staining showed that cilostazol treatment enhanced adiponectin expression in muscle cells; moreover, the expression was colocalized in ECs and SMCs (Figure 6A,B). In the sh-adipoR groups, the expression of adiponectin and the number of ECs and SMCs were lower than those of the sh-pLk group. This implied that adiponectin plays a pivotal role in modulating the angiogenic effects of cilostazol by binding to adipoRs. Furthermore, the relevant downstream signaling molecules of adiponectin/adipoRs, including the phosphorylation of AMPKα, ACC, Akt, and eNOS and the expression of SIRT1, were decreased in the sh-adipoR groups (Figure 6C,D), suggesting that the angiogenic effects of cilostazol were mediated by adiponectin, adipoRs, and the downstream pathway.

## 3. Discussion

**Figure 7:**
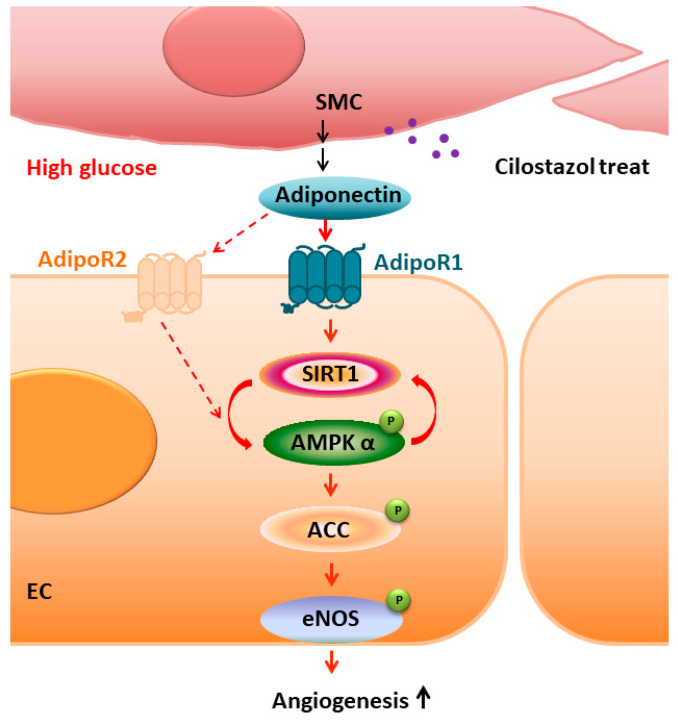
Schematic diagram shows the vascular angiogenic effects of cilostazol through stimulation of adiponectin secretion, adipoRs (mainly adipoR1), and the SIRT1 signaling pathway. ACC: acetyl-coenzyme A carboxylase; adipoR1: adiponectin receptor 1; adipoR2: adiponectin receptor 2; AMPK: adenosine monophosphate-activated protein kinase; EC: endothelial cell; eNOS: endothelial nitric oxide synthase; SIRT1: sirtuin 1; SMC: smooth muscle cell.

Our data, for the first time, demonstrated that cilostazol improved vascular angiogenic functions under diabetic conditions by increasing adiponectin secretion and activating the adipoRs/SIRT1/AMPK signaling pathway (Figure 7) both in vitro and in vivo.

A crosstalk between ECs and SMCs in regulating vascular functions may offer strategic insight into treating atherosclerotic disease, and the mode of action is probably via direct contact, eNOS-derived nitric oxide, extracellular matrix, extracellular vesicles, microRNAs, and other factors [28]. According to our study, cilostazol stimulates adiponectin secretion from SMCs but not ECs and enhances angiogenic effects through interaction with adipoRs in ECs. Therefore, cilostazol regulates adiponectin through crosstalk between ECs and SMCs via paracrine effects. Adiponectin is produced predominantly by adipose tissue [9], but whether cilostazol will also stimulate adiponectin secretion from adipocytes is still unknown. It remains poorly understood how cilostazol stimulates the secretion of adiponectin. Since cilostazol improves the response to ischemia in diabetic mice by a mechanism dependent on PPARγ [29] and since PPARγ agonists have demonstrated increased adiponectin in patients [30], we suppose that PPARγ may be a possible mechanism for increased adiponectin expression. However, this warrants further investigation.

The activation of AMPK is a well-known downstream effect of adiponectin [12,13] and plays a key role in modulating the energy-consuming anabolic pathway [31]. AMPK was reported to be indirectly involved in SIRT1 activation through the phosphorylation of GAPDH [32], and SIRT1 functions as an upstream regulator of liver kinase B1 (LKB1)/AMPK signaling [33]. Both AMPK and SIRT1 mutually regulate each other’s activities, a mechanism of action that is required for adiponectin-mediated cardiovascular protection [24]. Our data also showed that the AMPK/SIRT axis is required to promote the proliferation of HUVECs cocultured with cilostazol-treated HASMCs. Therefore, we speculated that cilostazol stimulates adiponectin secretion from SMCs and then interacts with adipoR1 to ameliorate angiogenesis through the AMPK/SIRT axis, ACC, and eNOS. On the other hand, reactive oxygen species (ROS) are also widely known to play an important role in modulating the effects of cilostazol [34]. Adiponectin has also been demonstrated protection against insulin resistance [35] and endoplasmic reticulum stress-induced apoptosis [36] via regulating ROS. The association between stimulating adiponectin and modulating ROS with cilostazol warrants further investigation.

Our previous data demonstrated that cilostazol improves angiogenesis by modulating AMPK/ACC in parallel with the cAMP/protein kinase A signaling pathway [6]. In this study, we further identified the role of adiponectin in these two parallel pathways of cilostazol. In a hindlimb ischemia model of diabetic mice, cilostazol increased adiponectin levels and the downstream signaling pathway, AMPK/SIRT, ACC, and eNOS, and subsequently improved angiogenesis. However, after the adipoR1 knockdown, all downstream pathways and blood flow recovery were attenuated to similar levels to those in mice without cilostazol treatment. We speculated that this pathway mediated by adiponectin plays a dominant and important role in the angiogenic effects of cilostazol.

In addition to the angiogenic effects, adiponectin is also a therapeutic target for insulin resistance, type 2 diabetes, and metabolic syndrome [10,37]. A meta-analysis indicated that type 2 diabetes risk was strongly associated with low levels of adiponectin [38]. The mechanism by which adiponectin improves glucose metabolism may be through AMPK, interleukin-6, tumor necrosis factor-α, inducible nitric oxide synthase, and PPARα [37]. However, adiponectin levels have also been reported to be strongly correlated with lipid profiles in patients with type 2 diabetes [39]. The possible mechanism for this may be due to the fact that adiponectin can promote apolipoprotein A-I/high density lipoprotein-mediated cholesterol efflux via ABCA1 [40]. Therefore, since cilostazol can promote the secretion of adiponectin, the beneficial effects of adiponectin promoted by cilostazol may be speculated.

## 4. Materials and Methods

### 4.1. Reagents

Fetal bovine serum (FBS), M199 medium, Opti-MEM I, 4,6-diamidino-2-phenylindole (DAPI), TRIzol reagent, and trypsin solution were purchased from Invitrogen (Carlsbad, CA, USA). Cilostazol, EX527 (a SIRT1 inhibitor), Griess-Romijn, crystal violet reagent, dimethyl sulfoxide, and anti-β-actin antibodies were purchased from Sigma–Aldrich (St. Louis, MO, USA). SRT 1720 (a SIRT1 activator) was purchased from Cayman Chemical (Ann Arber, MI, USA). The polymerase chain reaction (PCR) primers for adiponectin and GAPDH were purchased from Pollster Biotechnology (Taipei, Taiwan). Antibodies against adiponectin (#21613-1) was purchased from Proteintech (Rosemont, IL, USA). Antibodies against phosphorylated Akt (Ser437, #9271), Akt, phosphorylated AMPKα (Thr172, #2535), AMPKα (#2603), phosphorylated ACC (Ser79, #3661), ACC (#3676), and SIRT1 (#9475) were purchased from Cell Signaling Technology (Danvers, MA, USA). Antibodies against phosphorylated eNOS (Ser1177, #612393) and eNOS (#610297) were purchased from BD Transduction Laboratories (Franklin, CA, USA). Antibodies against adipoR1 (# sc-46749) and adipoR2 (# sc-46751) were purchased from Santa Cruz Biotechnology (Santa Cruz, CA, USA). Matrigel and rat monoclonal antibodies against murine CD31 (#557355) were purchased from BD Biosciences (Franklin Lakes, NJ, USA). Biotinylated rabbit anti-rat secondary antibodies, 3-amino-9-ethylcarbazole (AEC), and streptavidin–horseradish peroxidase (HRP) were purchased from Dako (Glostrup, Denmark). A 5-bromo-2-deoxyuridine (BrdU) kit was purchased from Roche Diagnostics (Mannheim, Germany).

### 4.2. Cell Culture

HUVECs were freshly isolated from human umbilical cord veins of newborn babies with previously described methods [41]. Informed consent was obtained from all the participating mothers prior to sample collection. Each patient provided written informed consent. The protocol for this study (ER-100-072) was approved by the Institutional Review Board of National Cheng Kung University Hospital. Cells were cultured in medium 199 containing 20% FBS, 25 pg/mL EC growth factor, 10 units/mL heparin, and 100 units/mL penicillin at 37 °C in a 5% CO_2_ and 95% humidity incubator. Cells cultured up to the third passage were used in all experiments.

Primary passage cryopreserved HASMCs were obtained from Cascade Biologics (Portland, OR, USA). Cells were plated in 75-cm^2^ flasks and incubated in M199 supplemented with 10% FBS and 5% smooth muscle growth supplement (Thermo Fischer Scientific, Waltham, MA, USA). Cells cultured up to passages 6 to 9 were used for experiments.

For this experiment, both cell lines were cultured with 25 mM of glucose to induce HG conditions.

### 4.3. Transwell Coculture Experiments

Coculturing of cells was performed using a Transwell system. Transwell chambers (0.4 μm pore diameter, Costar, Cambridge, MA, USA) were used to establish HUVEC and HASMC coculture models. HASMCs were seeded at a density of 2.5 × 10^3^ cells/cm^2^ in the lower chamber, and HUVECs were seeded at a density of 1 × 10^4^ cells/cm^2^ in the upper chamber. The upper and lower compartments were separated by a polycarbonate membrane to allow the free circulation of various cytokines and metabolites secreted by the cells. For preparation of the conditioned medium (CM), HASMCs were plated in 6-well dishes and grown until 90% confluence. Afterwards, the culture medium was incubated in M199 medium with 1% FBS, HG, and 100 μM of cilostazol for 2 days. 

### 4.4. Immunofluorescence Staining

Transwell membranes were fixed with 4% paraformaldehyde and permeabilized with 0.2% Triton X-100. The cells were then incubated overnight with primary anti-αSMA antibodies (Sigma-Aldrich, St. Louis, MO, USA) for SMCs and/or primary anti-CD31 antibodies (Dako, Denmark) for ECs. After the cells were washed with 0.1% Tween in PBS, they were incubated for 30 min with secondary Alexa Fluor 488-goat anti-rabbit antibodies (Invitrogen, Carlsbad, CA, USA). Nuclei were labeled with DAPI.

For ischemic muscle staining, samples were fixed with paraformaldehyde and embedded in paraffin. Sections were incubated with the following primary antibodies at 4 °C overnight: rabbit anti-CD31 (Abcam, Cambridge, UK) or rabbit anti-αSMA (Abcam, Cambridge, UK) and mouse anti-adiponectin antibodies (Proteintech, Rosemont, IL, USA). Then, sections were incubated with appropriate Alexa-Fluor-conjugated secondary antibodies (Thermo Fisher Scientific, Waltham, MA, USA) at room temperature for 30 min, and DAPI was used to label cell nuclei. Staining signals were captured using confocal microscopy (FV3000, Olympus, Tokyo, Japan).

### 4.5. Enzyme-Linked Immunosorbent Assay (ELISA)

HASMCs were seeded in a 96-well plate for 48 h. Thereafter, cells were cultured in M199 with 1% FBS, various concentrations of cilostazol, and 25 mM of glucose for 24 h. Adiponectin secreted into the culture medium was measured using a commercial ELISA kit (R&D Systems, Minneapolis, MN, USA). Assays were performed as recommended by the manufacturer.

Cocultured Transwell chambers were prepared and treated with cilostazol or vehicle in HASMCs layers (lower layers) or HUVECs (upper layers) alone in HG environments. After 24 h, media were collected from the two layers of the membrane (ECs and SMCs CM). Samples were assayed according to the methodology described above.

### 4.6. Short Hairpin RNA (shRNA) Transfection

HUVECs were transfected with 1–2 μg of plasmid DNA with Opti-MEM I medium and Lipofectamine 3000 (Invitrogen, Carlsbad, CA, USA) reagent for 15 min, according to the manufacturer’s instructions. All plasmids were purchased from the National RNAi Core Facility (Taipei, Taiwan). After transfection for 48 h, the cells were treated with various reagents in serum-free M199 medium. The oligonucleotide sequences of the shRNAs used were adipoR1 (5′-CCACTTCTATGGAGTCTCCAA-3′), adipoR2 (5′-GCTCTTCTCTAAACTGGATTA-3′), and AMPKα (5′CCTGGAAGTCACACAATAGAA-3′), SIRT1 (5′-CAGGTCAAGGGATGGTATTTA-3′) with nontarget RNA as a control.

### 4.7. Proliferation Assay

BrdU incorporation was analyzed using a cell proliferation ELISA kit (Roche Diagnostics, Mannheim, Germany) according to the manufacturer’s protocol. In the coculture system, HASMCs were seeded in a 24-well plate and maintained in M199 including 10% FBS and 25 mM of glucose until 80% confluence. HUVECs were seeded in the inserts (0.4–μm pore size) and grown in M199 with 20% FBS and 25 mM of glucose for 24 h. HASMCs were replaced with M199 medium including 1% FBS and treated with or without 100 μM of cilostazol for 24 h. The next day, HUVECs were added to 100 μM of BrdU and incubated at 37 °C for 4 h. The absorbance of the solution in each well was determined to be 570 nm using a Varioskan LUX Multimode Microplate Reader (Thermo Fischer Scientific, Waltham, MA, USA).

### 4.8. Migration Assay

The migration of HUVECs was tested with 8.0 µm Transwell inserts (Corning, Cambridge, MA, USA) as described previously [42]. HUVECs (1 × 10^5^ cells in 100 μL) were placed in the upper chamber with M199 medium supplemented with 1% FBS and 25mM of glucose, whereas HASMCs with or without 100 μM of cilostazol were placed in the lower chamber. After incubation at 37 °C for 24 h, the cells were fixed with 4% paraformaldehyde and stained with 0.5% crystal violet. Migrated cells were calculated using an Olympus CKX31 microscope (Tokyo, Japan) with Image-Pro Plus software (NIH, Littleton, CO, USA).

### 4.9. Matrigel Tube Formation

HUVECs (2 × 10^4^ cells/well) were plated on top of 200 μL of Matrigel Matrix (BD, 356,234) in a 48-well plate. Cell suspensions (2 × 10^4^) in 200 μL containing HASMCs –CM or M199 with 1% FBS and 25 mM of glucose were dispensed onto the Matrigel. After incubation for 6 h, the total tube area was quantified as the mean relative tube length obtained using image analysis with MacBioPhotonics ImageJ software (v1.51, National Institutes of Health, Bethesda, MD, USA).

### 4.10. Analysis of Apoptosis

The incidence of apoptosis was measured using a Cell Death Detection kit (Roche Diagnostics, Germany) according to the manufacturer’s protocol. Briefly, HUVECs were grown in the insert and were cocultured with HASMCs with or without 100 μM of cilostazol for 24 h (as above). Nucleosome fragmentation was assessed using a cell death detection ELISA kit. Experiments were performed four times.

### 4.11. Measurement of Nitric Oxide Production

Supernatants of HUVECs were collected after coculturing with HASMCs for 1 h. Exactly 100 μL of supernatant was mixed with 10 mL of Griess-Romijn reagent (100 mg/mL). The absorbance of the mixtures was spectrophotometrically measured at a wavelength of 540 nm.

### 4.12. RNA Isolation, Reverse Transcription, and Real-Time PCR

Total RNA was extracted using TRIzol reagent according to the manufacturer’s protocol and then reverse transcribed into cDNA using MultiScribe™ Reverse Transcriptase (Applied Biosystems, Waltham, MA, USA). Gene expression was quantitated using SYBR Green (Qiagen, Hilden, Denmark) with a Step One Plus real-time PCR system (Applied Biosystems, Waltham, MA, USA). The expression levels of each mRNA were calculated using the 2^−ΔΔCt^ method, and GAPDH was used as an internal control. The respective primers were adiponectin (forward: 5’-ACAGGAGATGTTGGAATGACAG-3’; reverse: 5’-CTGCCGTCATAATGATTCTGTT-3’) and GAPDH (forward: 5’-TGGCAACAATATCCACTTTACC-3’; reverse: 5’-AAGGTG AAGGTCGGAGTCAAC-3’).

### 4.13. Western Blot Analysis

HUVECs and HASMCs were cocultured in a 6 cm dish as above for 24 h. HUVECs were lysed using RIPA lysis buffer (Biotools, Taipei, Taiwan), and the protein concentration was determined by using a BCA Protein Assay Kit (Thermo Fisher Scientific, Waltham, MA, USA). Approximately 30 μg of protein samples was separated by 8–10% SDS/PAGE (polyacrylamide gel) and then transferred to PVDF membranes (Millipore, Burlington, MA, USA). Primary antibodies were used against the following targets: adipoR1, adipoR2, SIRT1, phosphorylated AMPK α (Thr172), AMPK-α, phosphorylated ACC (Ser79), ACC, phosphorylated eNOS (Ser1177 and Thr495), eNOS, phosphorylated Akt (Ser473), Akt, and β-actin. The membranes were exposed to HRP-conjugated secondary antibodies, and the blots were developed using enhanced chemiluminescence (PerkinElmer, CT, USA) followed by exposure to X-ray film (Fujifilm Medical, Tokyo, Japan). β-actin was used as an internal control.

### 4.14. Diabetic Mice and Murine Hindlimb Ischemia Model

These animal experiments were performed at the College of Medicine, National Cheng Kung University (Tainan, Taiwan). All experiments performed on the animals were approved by the Institutional Animal Care and Use Committee, National Cheng Kung University, Tainan, Taiwan (IACUC number: 100024). All methods were performed in accordance with relevant institutional and ARRIVE guidelines [43] and regulations. Eight-week-old male ICR mice were intraperitoneally injected with STZ, and 150 mg/kg in citrate buffer (pH 4.5) was used to induce experimental hyperglycemia. After 2 weeks, plasma glucose levels after overnight fasting were measured to confirm that these mice were hyperglycemic.

Diabetic mice were anesthetized with an intraperitoneal injection of pentobarbital (80 mg/kg) followed by ligation of the proximal segment of the right femoral artery, as previously described [5,44]. Cilostazol (10 mg/kg of body weight) was injected intraperitoneally 30 min prior to induction of ischemia and then twice a day at the same dose from days 2 to 7. Control animals received equivalent volumes of normal saline. The mice were euthanized by inhalation of excessive carbon dioxide 28 days after surgery.

### 4.15. Knockdown of adipoR1 and adipoR2 in the Hindlimb

The constructs of sh-adipoR1, sh-adipoR2 and sh-pLk0.1 were provided with 10% glucose solution. Plasmid and in vivo jetPEI (Illkirch, France) reagent were dissolved and prepared according to the optimized manufacturer’s protocol (N:P = 10). Exactly 100 μL of transfection complexes was injected intramuscularly into multiple sites on the hindlimbs on days 0, 7, 14, and 21 after inducing hindlimb ischemia.

### 4.16. Laser Doppler Flow Imaging

Blood perfusion in hindlimb blood flow was measured using a laser Doppler perfusion imaging analyzer (Moor Instruments, Devon, UK) preoperatively and postoperatively on days 3, 7, 14, 21, and 28 after surgery. The digital color-coded images were analyzed to quantify the blood flow in the region from the knee joint to the toes. The perfusion rate (%) was calculated as the ratio of blood flow on the ipsilateral side compared with the contralateral side.

### 4.17. Measurement of Capillary Density in Ischemic Limbs

Muscle tissue from the ischemic gastrocnemius muscle was harvested, fixed with methanol, embedded in paraffin, and cross-sectioned for histological immunostaining. Primary antibodies against CD31 (1:50) for capillary detection were incubated at 4 °C overnight. Sections were then incubated with biotinylated rabbit anti-rat secondary antibodies (1:500) for 30 min. Streptavidin-HRP was added for 10 min, and color development was performed with the addition of the AEC substrate. Five sections were randomly studied from each mouse. The slides were counterstained with hematoxylin, and digital images were captured using an Olympus IX71 microscope. Capillary density was quantified by counting the mean number of capillaries, as revealed by positive expression of CD31 in ECs.

### 4.18. Enumeration of Circulating Progenitor Cells

Hematopoietic progenitor cells (CD34^+^CD45^dim^) were enumerated according to the International Society of Hematotherapy and Graft Engineering (ISHAGE) guidelines. Briefly, 50 μL of whole blood obtained from mouse tail veins was incubated with phycoerythrin-conjugated anti-CD34 and FITC-conjugated anti-CD45 monoclonal antibodies (BD Pharmingen, Franklin Lakes, NJ, USA) in a TruCOUNT tube (BD Pharmingen, Franklin Lakes, NJ, USA) following the manufacturer’s instructions. CD34^+^CD45^dim^ cells were then analyzed and quantified using a FACSCanto flow cytometer according to the ISHAGE sequential gating strategy.

### 4.19. Statistical Analysis

All data are expressed as the mean ± standard error of the mean (SEM), and each point represents the average of at least 3 separate experiments. Statistical analysis was performed using Student’s t-test to compare the statistical significance of multiple groups. Statistical significance was tested at *p* < 0.05. 

## 5. Conclusions

In conclusion, cilostazol improves HG-induced endothelial dysfunction in vascular ECs and enhances angiogenesis in diabetic mice by upregulating the expression of adiponectin/adipoRs and its SIRT1/AMPK downstream signaling molecules. The results suggest that cilostazol provides therapeutic benefits for the treatment of diabetic patients with ischemic disease.

## Data Availability

Not applicable.
